# Expression Patterns for *TETs*, *LGR5* and *BMI1* in Cancer Stem-like Cells Isolated from Human Colon Cancer

**Published:** 2019

**Authors:** Nader Atlasy, Fardin Amidi, Keywan Mortezaee, Mohammad Sadegh Fazeli, Seyed Javad Mowla, Fatemeh Malek

**Affiliations:** 1. Department of Anatomy, Faculty of Medicine, Tehran University of Medical Sciences, Tehran, Iran; 2. Department of Anatomy, Faculty of Medicine, Kurdistan University of Medical Sciences, Sanandaj, Iran; 3. Department of Surgery, Imam Khomeini Hospital, Tehran University of Medical Sciences, Tehran, Iran; 4. Department of Genetics, Faculty of Life Sciences, Tarbiat Modares University, Tehran, Iran

**Keywords:** Colon, Flow cytometry, Molony murine leukemia virus, Neoplastic stem cells

## Abstract

**Background:**

Colon tumor is generated and maintained by a small subset of chemo-resistant cancer cells known as Cancer Stem-like Cells (CSCs) that are able to self-renew and differentiate into various cell types within the cancer milieu. CSCs are identified through expression of CD133 that is the most important surface marker of these cells. Epithelial Cell Adhesion Molecule (EpCAM) is another colon CSCs marker. Other markers that are probably involved in colon tumorigenesis are Leucine-rich repeat-containing G-protein-coupled Receptor 5 (LGR5), B cell-specific Moloney murine leukemia virus insertion site 1 (BMI1) and Ten-Eleven Translocations (TETs).

**Methods:**

Here, mRNA expression rates of *LGR5*, *BMI1* and *TETs* were surveyed by real-time PCR. After collection and digestion, colon samples were used to isolate CD133 and EpCAM positive CSCs through evaluation of AC133 EpCAM by Magnetic Activated Cell Sorting (MACS) and flow cytometry. Real-time PCR was carried out for assessing expressions of *LGR5*, *BMI1* and *TETs*.

**Results:**

High expressions for *LGR5, BMI1, TET1* and *TET2* in the CD133 and EpCAM positive CSCs (p≤0.05 *vs.* non-CSCs) were found. *TET3*, however, showed no significant changes for mRNA expression in the CSCs.

**Conclusion:**

In conclusion, high mRNA expressions for *LGR5, BMI1, TET1* and *TET2* in the CD133 and EpCAM positive CSCs may be a useful criterion for better identification of the cells involved in colon cancer in order to specify therapeutic targets against this type of cancer.

## Introduction

Colorectal Cancer (CRC) is the second most common cancer diagnosed in women and the third most common cancer in men 1. CRC is identified to have subpopulation of highly resistant Cancer Stem-like Cells (CSCs) 2; CSCs are the minority and undifferentiated population located at the top of the tumor and involved in the re-establishment of tumor heterogeneity, while their progeny are the majority and terminal differentiated cells located at the base of the tumor and they do not contribute to tumor growth 3,4. Therefore, targeting CSCs may provide a therapeutic approach for managing metastatic disease 5.

CD133 (also called prominin-1) is the most important surface marker of CSCs 6 that is related to the tumorigenicity, poor prognosis and disease progression 2. Epithelial Cell Adhesion Molecule (EpCAM) is another colon CSCs marker that has been reported to be overexpressed in CRC and has an essential role in cancer prognosis and pathogenesis 1.

B cell-specific Moloney murine leukemia virus insertion site 1 (BMI1) is a marker of intestinal stem cells able to label quiescent stem cells. G-protein-coupled receptor 5 (LGR5) is also upregulated in CRCs 7. LGR5 may play an essential role in the prognosis and progression of CRC, and may be considered as a potential new therapeutic approach for targeting CRC. It may also be regarded as a potential marker for colorectal CSCs 8. Melo *et al* found that selective LGR5^+^ cell ablation restricts primary colon tumor growth, but does not result in tumor regression 5.

Variation of Ten-Eleven Translocation (TET) proteins (TET1, TET2 and TET3) is common in human cancers 9. TET enzymes affect cancer cell activity and presumably alter genomic 5hmC and 5mC patterns. In addition, 5mC oxidation seems to be a step for various pathways of activating DNA demethylation. This is probably essential for induction of global hypomethylation that occurs during cancer development and progression. Much has been documented regarding this interesting pathway for modification of DNA in recent years, but there is a need for more research so as to identify possible roles for TET proteins in gene regulation during carcinogenesis 10. Moreover, possible differences in the rate of expressions between various types of *TETs* remain to be clarified. Here, mRNA expressions were analyzed for *TETs*, *LGR5* and *BMI1* in CSCs isolated from human colon cancer samples.

## Materials and Methods

### Specimen preparation

Tumor samples and their matched normal tissues were collected from 14 patients diagnosed with colon adenocarcinoma. Samples were thoroughly washed three to four times in cold Phosphate-Buffered Saline (PBS pH=7.4, Gibco, Carlsbad, CA, USA) containing penicillin/streptomycin and amphotericin B (Invitrogen, Carlsbad, CA, USA) followed by incubation in Dulbecco's Modified Eagle's Medium (DMEM)/F12 (Invitrogen, Grand Island, NY) overnight at 4*°C* in dark. Patients exposed to chemo/radio therapy prior to surgery were not allowed to be registered for this experiment. Additional tumor and matched normal tissue fragments were kept in liquid nitrogen. For mechanical and enzymatic digestion, tissue specimens were first minced into 2 *mm*^2^ fragments, and then digested using 1.5 *mg/ml* collagenase IV (Invitrogen, Carlsbad, CA, USA), 20 *μg/ml* hyaluronidase and 40 *μg/ml* DNase I (Sigma Chemical Co., St. USA) for 1 *hr* at 37*°C* with pipetting every 10 *min*. Prior to inclusion in the study, all patients received informed consent that meets Research Ethics criteria of Tehran University of Medical Sciences and the principles of the Declaration of Helsinki and Good Clinical Practice Guidelines.

### Magnetic activated cell sorting (MACS)

AC133 and EpCAM isolation kits (Miltenyi Biotec, Bergisch-Gladbach, Germany) were used for cell isolation according to the manufacturer's protocol. AC133 antibody is frequently used for isolation of CSCs through detecting a glycosylated epitope of CD133 on these cells 11. In brief, up to 10^8^ cells were isolated from colon cancer samples and their matched normal specimens. Samples were labeled with mouse anti-AC133 microbeads conjugated antibody (1:10) for 30 *min* at 4*°C* and washed in the MACS buffer. The cells were loaded onto the MACS MS column placed on the magnetic cell separator. AC133^+^ cells were attached to the column, while AC133^-^ passed through it as negative fraction. Positive cells were then resuspended in the MACS buffer. The same procedure was performed to isolate EpCAM^+^ cells by using mouse anti-EpCAM microbeads conjugated antibody (1:10).

### Flow cytometry

10^5^ cells were suspended in 50 *μl* PBS containing 4% bovine serum albumin (BSA, Sigma Chemical Co., St. Louis, MO, USA) and labeled with mouse anti-AC133-APC and anti-EpCAM antibodies (1:10, 30 *min*, 4*°C*). The cells were washed with PBS and fixed in 4% paraformaldehyde (PFA, Sigma, CA, USA) at room temperature. Cells were then resuspended in PBS and examined by FACS Calibur Flow Cytometer (Becton Dickinson, San Jose, Canada).

### Real-time PCR

Total RNA was extracted using extraction kit (GeneAll Biotechnology, Seoul Republic of Korea), and RNase-free DNase I (Cinnagen, Tehran, Iran) was added to thermal cycler for 30 *min* at 37*°C* in order to remove genomic DNA contamination. Then, 1 *μg* of extracted mRNA was reverse transcripted to cDNA using cDNA Synthesis Kit (Thermo Scientific, Vilnius, Lithuania). Real-time PCR was performed using specific gene primers, cDNA and additional PCR reagents (dNTP, polymerase, magnesium and buffer; 5×HOT FIREPol® EvaGreen® qPCR Mix Plus [ROX] 1 *ml* 08-24-00001 Solis Bio Dyne, Tartu, Estonia) on three-color real-time PCR machine (Applied Biosystems Step One, CA, USA). Firstly, samples were incubated for initial activation of polymerase at 95*°C* for 15 *min*. Samples were then denatured at 95*°C* for 15 *s*, annealed at 60*°C* for 20 *s* and elongated at 72*°C* for 20 *s*. Relative quantification was carried out using the 2^−∆CT^ technique that includes normalization of the data to β-actin and further comparison of the fold change calculation to the non-CSCs.

Primers were as follows: *LGR5*, F “GGAAATCATGC CTTACAGAGC” and R “CCTGGGGAAGGTGAAC ACT”; *BMI1*, F “CTGGTTGCCCATTGACAGC” and R “CAGAAAATGAATGCGAGCCA”; *TET1*, F “AAT GGAAGCACTGTGGTTTG” and R “ACATGGAGC TGCTCATCTTG”; *TET2*, F “TTGGACTTCTGTGCT CATGC” and R “CATCCTCAGGTTTTCCTCCA”; *TET3*, F “TCGGAGACACCCTCTACCAG” and R “C TTGCAGCCGTTGAAGTACA”; and *β-actin*, F “TC CCTGGAGAAGAGCTACG” and R “GTAGTTTCG TGGATGCCACA”. F=Forward and R=Reverse.

### Statistical analysis

Statistical analysis was carried out using SPSS 16 and independent samples T-Test to evaluate significant differences between groups. Quantitative variables were presented as mean±standard deviation (SD), and p≤ 0.05 was considered statistically significant.

## Results

### MACS and flow cytometry findings

**Figure 1.**
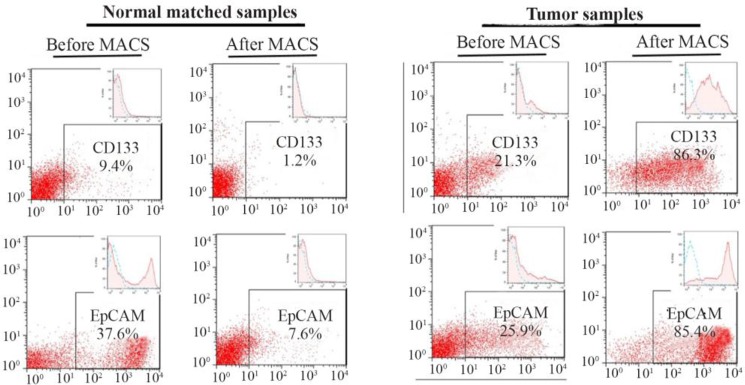
Flow cytometry assay of tumor cells and their matched normal cells isolated using a Magnetic-Activated Cell Sorter (MACS). Tumor cells showed high rates of expressions for cancer-stem like cell markers (*i.e*. CD133 and EpCAM). Results are presented as mean±SD (n=8).

CD133 and EpCAM markers were assessed by MACS and flow cytometry. Tumor cells showed high rates of CD133 and EpCAM expressions with 85.8±0.8 and 85.1±0.4, respectively. Normal cells from the same patients, on the other hand, had low rates of expressions for CD133 and EpCAM with 1.3±0.1 and 8.1± 0.9, respectively. The rates of expressions for both markers were significant in the tumor cells compared to their matched normal cells (p≤0.05) (Figure 1).

### Real-time PCR

**Figure 2.**
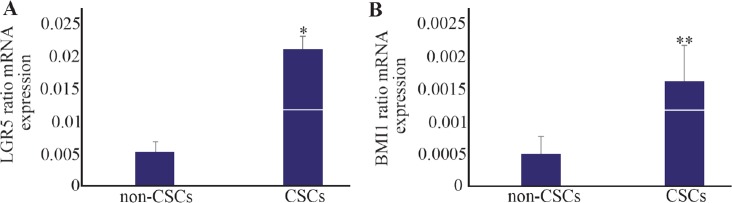
Relative mRNA expressions for leucine-rich repeat-containing G-protein-coupled receptor *(LGR5)* and B cell-specific Moloney murine leukemia virus insertion site 1 *(BMI1)* analyzed by real-time RT-PCR. A) LGR5 had high level of expression in the cancer stem-like cells *(CSCs)*. B) *BMI1* showed a similar pattern with higher rate of expression in the CSCs compared with the non-CSCs. * p≤0.001 and ** p≤0.03 *vs*. non-CSCs. Results are presented as mean±SD (n=14).

Real-time PCR was performed to evaluate expression rates for various CSCs markers. *LGR5* showed a higher rate of expression with 4-fold increase in the CSCs compared with the non-CSCs. The mRNA levels for CSCs and non-CSCs were 0.02±0.002 and 0.005± 0.001, respectively (p≤0.001) (Figure 2A).

Similarly, *BMI1* had a higher rate of expression with 4-fold increase in the CSCs compared with the non-CSCs. The mRNA expressions for CSCs and non-CSCs were 0.0016±0.0005 and 0.0005±0.0002, respectively. The levels were significant in the CSCs compared with non-CSCs (p≤0.03) (Figure 2B).

**Figure 3.**
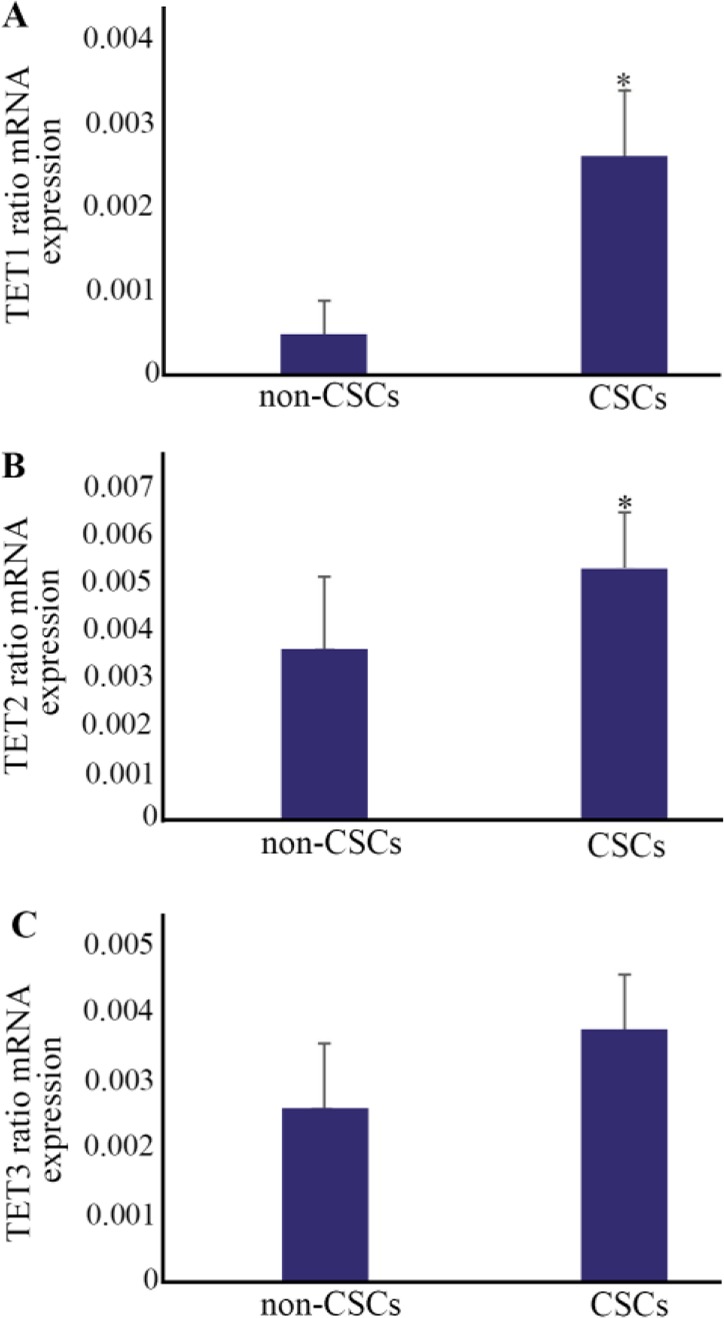
Relative mRNA expressions of Ten-Eleven Translocation *(TET)* enzymes (*TET1*, *TET2* and *TET3*) assessed by real-time RT-PCR. A) There was a noticeable increase for *TET1* expression in cancer stem-like cells (CSCs). *p≤0.004 *vs*. non-CSCs. B) *TET2* showed significant changes in levels of mRNA expression between CSCs and non-CSCs. *p≤0.02 *vs*. non-CSCs. C) *TET3*, however, showed no considerable differences in levels of mRNA expression between CSCs and non-CSCs. Results are presented as mean±SD (n=14).

Expression patterns of *TET1*, *TET2* and *TET3* were evaluated in the CSCs. *TET1* showed about 7-fold increase in the rate of expression in the CSCs with 0.0036±0.0007. The expression rate of *TET1* for non-CSCs was 0.0005±0.0004. The levels were significant in the CSCs compared with non-CSCs (p≤0.004) (Figure 3A).

*TET2* showed about 1.5-fold increase in the rate of expression in the CSCs with 0.0053±0.001. The expression rate of *TET2* for non-CSCs was 0.0036±0.001. The levels were significant in the CSCs compared with non-CSCs (p≤0.02) (Figure 3B).

Similarly, *TET3* had about 1.5-fold increase in the expression rate of the CSCs with 0.0038±0.0008. The rate of *TET3* expression in the non-CSCs was 0.0026± 0.0009. The levels were not considerable in the CSCs, as compared with the non-CSCs (p≤0.59) (Figure 3C).

## Discussion

In the current study, high expression of *CD133* was found in the CSCs isolated from human colon cancer. CD133 is a known stem cell marker that is widely used to identify colon CSCs 1. Saigusa *et al* referred to an increase in the gene and protein expressions for CD133 in patients with rectal cancer and also a line between CD133 expression with poor prognosis and distant recurrence 12. Kemper *et al* also attested an association between *CD133* expression with poor survival in patients suffering from CRC 13. On the other hand, Gazzaniga *et al* showed that *CD133* expression in circulating tumor cells harvested from peripheral blood from metastatic CRC patients had no association with overall outcome in the patients 14. Despite having a contradictory single report, most of the studies reinforce the idea of the potential application of CD133 as a prognostic marker for colon CSCs 1. Therefore, a high rate of *CD133* expression in the cells harvested from tumor samples subsequent to the second MACS indicates the collected cells obtained high levels of purity.

Expression of EpCAM is considered as a marker for detection of CRC 15. This marker is associated with proliferation 16 and metastasis 17 in cancer cell. In one report, however, a negative link between this marker with cancer cell proliferation has been documented 18. Due to most of the studies identified EpCAM as a prognostic marker for cancer progression, it can be assumed that high expression for EpCAM in the tumor cells may be a useful criterion for specific identification of the CSCs within the tumor milieu.

Overexpression of *LGR5* was found in CSCs. LGR5 is a marker gene for detection of CSCs in colon cancer 19,20, indicating differentiation capacity and self-renewal for LGR5^+^ tumor cells 20. High activity for LGR5^+^ cells is at the crypt base 20 where CSCs are presumably located 21. High level for LGR5 in CRC is also positively associated with histological grade and invasiveness 8. Higher levels for mRNA and protein expressions of *LGR5* in primary colon cancer compared with normal tissues in both animal and human 22 have also been reported to reflect shorter survival span in both models 22 so that selective LGR5 positive cell ablation limits primary colon tumor growth, but this does not lead to tumor regression. Instead, tumors are kept by proliferative LGR5 negative cells that continuously try to replenish the LGR5 positive CSC pool, resulting in rapid re-initiation of tumor growth upon treatment cessation 5.

BMI1 is an inducer of cancer cell migration and invasion 23. BMI1 is required for tumor growth maintenance in human CRC cells 24 in which its inhibition results in tumor growth arrest 25. BMI1 is also identified as a stem cell marker 23 and an important oncogene for promoting self-renewal of colon CSCs 23. Data from this work also showed significant expression of *BMI1* in CSCs compared with non-CSCs. Lower expression for *BMI1* was found than the expression for *LGR5* in CSCs. To illustrate, BMI1 was initially identified through its ability for labeling quiescent intestinal stem cells 26, but LGR5 was identified in relation with rapid cycling cells 27. Therefore, it is conceivable to speculate the existence of more proliferative and active LGR5 and CD133 positive CSCs in human colon cancer than the existence of quiescent BMI1 positive cells.

Expression rates of *TET*s have also been surveyed in this work. Significant expressions of *TET1* and *TET2* in CSCs were observed. *TET3,* however, showed no significant changes between the two types of cells examined in the present study. Data about expression rate of *TETs* in CSCs is so limited, and most of the knowledge in this regard has come from studies on Embryonic Stem Cells (ESCs) or on colon tissue tumor in general. *TET1* downregulation has been shown to promote cancer invasion and metastasis 28. In ESCs, TET1 was found to play an essential role in their self-renewal 29. However, in a study performed by Hu *et al*, ESCs lacking all three *TET* genes seemed normal in pluripotency and self-renewal 30. Neri *et al* noticed that downregulation of *TET1* in colon tumor in mice was not only associated with tumor malignancy and progression but also connected to tumor initiation and growth 28. It is still unclear why CSCs over express *TET1* and *TET2* in human subjects and further studies are required to investigate the reason.

## Conclusion

High rates of mRNA expressions for *LGR5*, *BMI1*, *TET1* and *TET2* in the CD133 and EpCAM positive CSCs of this study may be regarded as a useful criterion for detecting these cells from their progeny. When the cells are well identified, therapeutic protocols will be applied more specifically for these cells, as it has been done so far for glioblastoma 31 and ovarian cancer using melatonin 32.
